# Bioinformatics identify the role of chordin-like 1 in thyroid cancer

**DOI:** 10.1097/MD.0000000000032778

**Published:** 2023-02-03

**Authors:** Jia-Wei Yu, Rui Pang, Bo Liu, Liang Zhang, Jie-Wu Zhang

**Affiliations:** a Department of Head and Neck Thyroid, Harbin Medical University Cancer Hospital, Harbin, Heilongjiang, China.

**Keywords:** CHRDL1, co-expression, immune, thyroid cancer

## Abstract

The abnormal expression of chordin-like 1 (CHRDL1) is identified in many cancers, while the effect of CHRDL1 in thyroid cancer (THCA) remains unclear. The University of California Santa Cruz, Gene Expression Profiling Interactive Analysis, University of Alabama at Birmingham Cancer, and Gene Expression Omnibus database (GSE33570, GSE33630, and GSE60542) were used for determining the mRNA and methylation expression of CHRDL1 in tumor and normal tissues. Human Protein Atlas was used for exploring the protein expression level of CHRDL1. The genes correlated to CHRDL1 were assessed by cBioPortal database. The prognostic value of CHRDL1 was evaluated through Kaplan–Meier method, cox regression, and nomogram analysis. Kyoto Encyclopedia of Genes and Genomes, Gene Ontology, and gene set enrichment analysis were used for predicting potential function of CHRDL1. The relationship between CHRDL1 and immune cell infiltration was determined by Pearson method. The downregulated mRNA and protein expressions of CHRDL1 were identified in THCA through the analysis of data from The Cancer Genome Atlas, Gene Expression Omnibus, and Human Protein Atlas database. The survival analysis showed that the CHRDL1 expression significantly affected disease-free interval (DFI) and progression-free interval, and CHRDL1 was an independent predictor of DFI. Besides, we found that C-C motif chemokine ligand 21 could significantly affect DFI time when it was co-expressed with CHRDL1. Additionally, the function of CHRDL1 was enriched in cell migration, apoptosis, and immune cell receptor. The downregulated expression of CHRDL1 was observed in THCA and caused poor prognosis. CHRDL1 may be involved in signal pathway related to cancer development and immune response, which suggested it could be a potential biomarker.

## 1. Introduction

Thyroid cancer (THCA) is a common endocrine malignancy ranking ninth for incidence in all cancer types globally, but it has low mortality.^[1]^ There are 4 common subtypes of THCA. Papillary thyroid cancer and follicular thyroid cancer are 2 well-differentiated thyroid tumors (DTC), which account for most thyroid cancer cases and are related to positive prognosis and high survival rates.^[2,3]^ However, some DTC developed distant metastases have a poor prognosis.^[4]^ In addition, anaplastic thyroid cancer and medullary thyroid cancer are 2 subtypes of less-differentiated thyroid tumors with poor prognosis.^[5,6]^ Although chemoradiation is the main treatment for anaplastic thyroid cancer,^[7]^ there was still a risk of recurrence for patients accompanied by the chemoresistant,^[8]^ which was closely related to the early detection of THCA.^[9]^ Thus, it is essential to explore the molecular mechanisms and identified the key biomarkers of THCA.

Chordin-like 1 (CHRDL1) is one of the secretory proteins.^[10]^ Actually, CHRDL1 plays a vital role in the development of many cancers.^[2]^ Previous studies showed that CHRDL1 usually had a downregulated expression and could reduce the ability of cell proliferation and migration in many tumors.^[11,12]^ Moreover, CHRDL1 has been identified as a potential biomarker for cancer prognosis, including gastric cancer (GC) and THCA.^[13,14]^ Besides, the mechanisms of CHRDL1 in some cancers have been reported. Wang et al found that CHRDL1 expression was regulated by hsa-microRNA-204 expression, which affected the invasion and proliferation of GC cells.^[15]^ Meanwhile, Wang et al found that hsa-miR-204/CHRDL1 axis also affected the prognosis of breast cancer (BC).^[16]^ Cyr-Depauw et al^[17]^ claimed that CHRDL1 could inhibit the migration and invasion of BC cells to alleviate the prognosis of BC through inhibiting the bone morphogenetic protein 4 (BMP4) signal. Furthermore, the negative effect of CHRDL1 in BMP4 expression on inhibiting the dedifferentiation of diffuse intrinsic pontine glioma has also been reported.^[18]^ In addition, further research found that CHRDL1 expression was downregulated by hypermethylation and the downregulated CHRLD1 expression promoted BMP4 ligation to BMP receptor II in GC, which caused the aberrant activation of protein kinase B (AKT), extracellular regulated protein kinases and β-catenin and further promoted the proliferation and metastasis of GC cells.^[11]^ However, the mechanisms of CHRDL1 in the prognosis of THCA are unknown.

**Figure 1.**
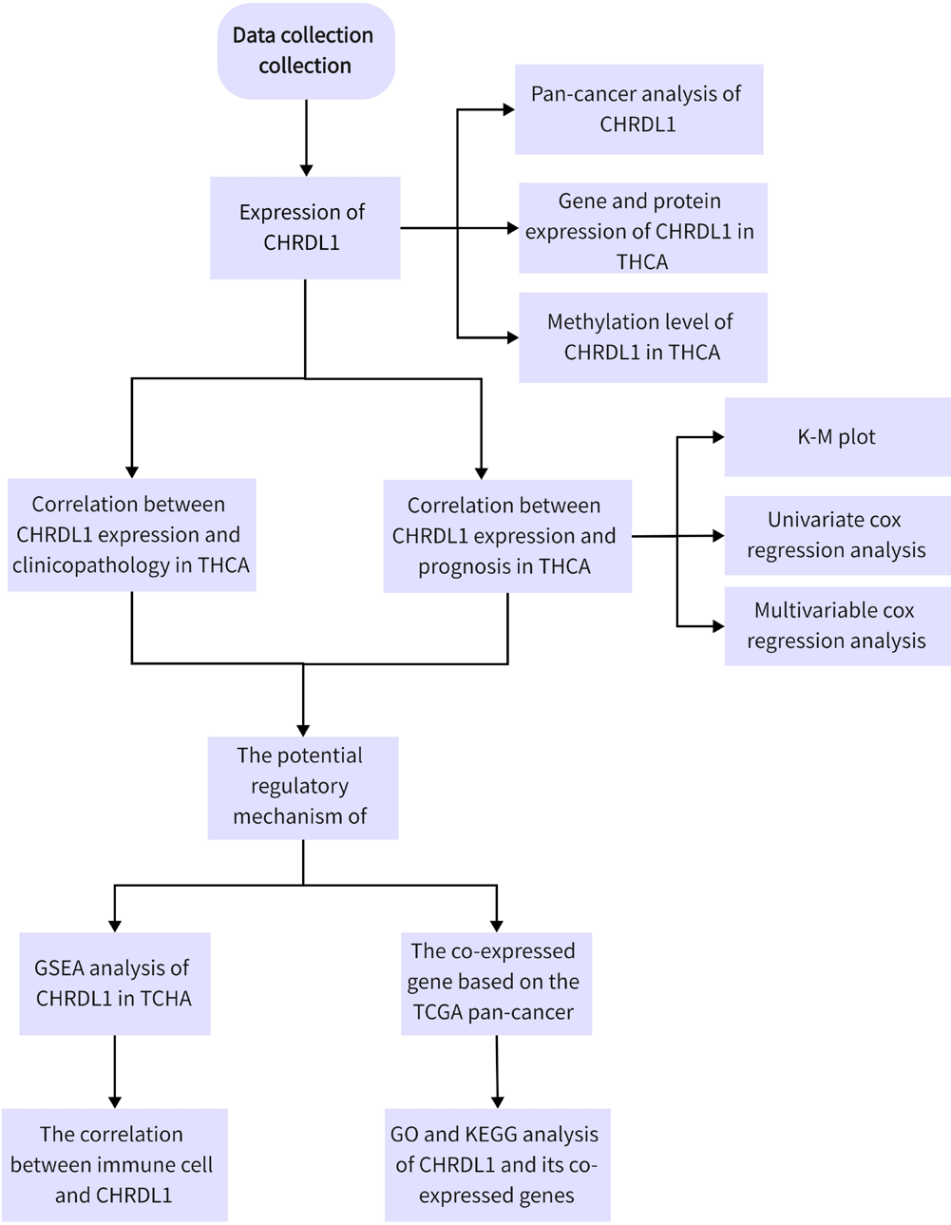
The flow chart of the identification of the CHRDL1 function in THCA. CHRDL1 = chordin-like 1, THCA = thyroid cancer.

Although the abnormal expression of CHRDL1 was reported in THCA,^[14,19,20]^ the mechanisms of CHRDL1 remain unclear. Thus, in this study, we collected CHRDL1 expression and survival data from The Cancer Genome Atlas (TCGA) database and further determine the role of CHRDL1 in the development of THCA through bioinformatics analysis. The research progress is shown in Figure 1.

## 2. Methods

### 2.1. University of California Santa Cruz (UCSC) xena database analysis

UCSC xena database (https://xenabrowser.net/) is a public and widely used biological database which is established and maintained by UCSC. It provides many genome data, containing gene expression and phenotype. We downloaded the gene expression RNA seq of CHRDL1 and corresponding clinical information of THCA patients, including age, gender, race, M stage, N stage, T stage, pathologic stage and survival time. The CHRDL1 were divided into high-expressed group and low-expressed group with optimal truncation. Then, these data were analyzed by R software (version 3.6.3, https://cran.r-project.org/bin/windows/base/old/3.6.3/) for further study.

### 2.2. Gene Expression Profiling Interactive Analysis (GEPIA) database analysis

GEPIA (http://gepia.cancer-pku.cn/) is a web-based tool. It provides differential expression analysis, multiple gene comparison, survival analysis, similar gene detection, correlation analysis and principal component analysis based on TCGA and genotype-tissue expression data. In our study, the mRNA expression of CHRDL1 in pan-cancer was also analyzed in GEPIA.

### 2.3. Gene Expression Omnibus (GEO) database

GEO (https://www.ncbi.nlm.nih.gov/geo/) is a public functional genomics data repository including and providing gene expressions based on the high-throughput sequencing data. In this study, 3 GEO datasets including GSE33570, GSE33630 and GSE60542 were selected to verify the mRNA expression of CHRDL1 in THCA and normal tissues.

### 2.4. The Human Protein Atlas (HPA) database analysis

The HPA (https://www.proteinatlas.org/) is a Swedish-based program which provides public and free resources of protein information in approximately 36 tissues. Furthermore, the protein distribution in tissues and cells could be identified through immunohistochemistry (IHC) staining images in the HPA database. In our study, we compared the protein expression of CHRDL1 between THCA and normal samples through IHC staining images.

### 2.5. The University of Alabama at Birmingham Cancer (UALCAN) data analysis portal

The UALCAN network (http://ualcan.path.uab.edu) is a comprehensive, user-friendly web resource for analyzing cancer data with mass data from TCGA, Clinical Proteomic Tumor Analysis Consortium and Children’s Brain Tumor Tissue Consortium. Besides, it also provides plenty of data about the expressions, correlated genes and methylation levels of cancer genes. It is helpful for exploring the biomarkers of some cancers. In our study, the data of CHRDL1 methylation level in THCA and normal samples were obtained in UALCAN.

### 2.6. cBioPortal database analysis

The cBioPortal for Cancer Genomics (http://cbioportal.org) is an available and open resource providing visual and multidimensional genomics data of 225 cancers.^[21]^ In our study, the genes positively and negatively correlated to CHRDL1 in THCA (TCGA Pan-Cancer Atlas) were obtained from the cBioPortal database and then they were subjected to the further enrichment analysis.

### 2.7. Gene Ontology (GO) term, Kyoto Encyclopedia of Genes and Genomes (KEGG) pathway enrichment analysis, and gene set enrichment analysis (GSEA)

GO enrichment analysis is an international method to describe the function of genes and proteins. It includes 3 categories, namely, biological process, cellular component and molecular function. KEGG is used for understanding biological functions and pathways. GSEA is a software co-developed by UC San Diego and Broad Institute and used for determining the role of genes in phenotypes through evaluating the trend of genes and phenotypes in a specific gene set. R software cluster Profiler package was used for KEGG and GO enrichment analysis of CHRDL1 and its co-expressed genes. GESA was performed by the GSEA software using a gene set database (c2.cp.kegg.v2022.1.Hs.symbols.gmt). The *P* < .05 and false discovery rate (FDR) of <0.25 were considered statistically significant.

### 2.8. Survival analysis

The correlations between the CHRDL1 expression and the prognosis including overall survival, disease-specific survival, disease-free interval (DFI), and progression-free interval (PFI) in THCA were examined using Kaplan–Meier (K–M) survival analysis. Then, we evaluated the prognosis value of CHRDL1 and clinical characteristics (age, gender, race, M stage, N stage, T stage, and pathologic stages) in THCA through univariate and multivariate cox regression. Furthermore, the nomogram analysis based on the results of cox regression was used for exhibiting the contribution of covariates in THCA. Besides, we assessed the connection between 5 co-expressed genes of CHRDL1 and prognosis though K–M survival analysis.

### 2.9. Statistical analysis

The statistical analysis was conducted by SPSS and R software. According to the results of Shapiro–Wilk test and quantile–quantile (Q–Q) plot, the parameter test is used to test the data conforming to the normal distribution, while the nonparametric test is used to test the data that does not conform to normal distribution. For quantitative data, the comparison between 2 groups was evaluated by independent sample *t* test, paired sample *t* test, Mann–Whitney *U* test or the Wilcoxon signed rank, while the comparison among 3 and 4 groups was evaluated by 1-way analysis of variance or Friedman test followed by post hoc comparisons. The survival difference between 2 groups was compared by log-rank test or life table methods. Besides, correlation between 2 quantitative variables was analyzed by Pearson test or Spearman test. *P* < .05 was considered as significant difference.

## 3. Results

### 3.1. The analysis of CHRL1 expression in cancers

**Figure 2.**
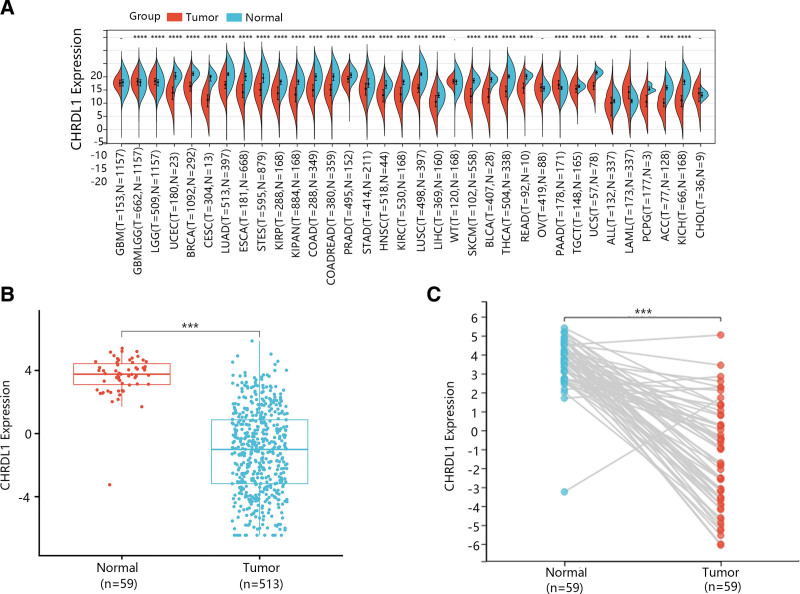
The mRNA expression of CHRDL1 in cancers. (A) The pan-cancer analysis of CHRLD1 in 34 cancers from TCGA and GTEx. (B) The expression of CHRDL1 in THCA and normal tissues was detected according to the independent samples from TCGA database. (C) The expression of CHRDL1 in THCA and normal tissues was detected according to the paired samples from TCGA database. ^*^P < .05, ^**^ *P* < .01, ^***^P < .001. CHRDL1 = chordin-like 1. GTEx = genotype-tissue expression, TCGA = the cancer genome atlas, THCA = thyroid cancer.

The CHRDL1 expressions between kinds of cancers and normal tissues were determined by the analysis of TCGA and genotype-tissue expression data. According to Figure 2A, the expression of CHRDL1 was markedly downregulated in most cancers but upregulated in glioma, brain lower grade glioma, pancreatic adenocarcinoma, acute myeloid leukemia and thymoma. As shown in Figure 2B, the CHRDL1 expression was evidently downregulated in THCA tumor tissues (*P* < .001). Besides, it was obvious that mRNA expression of CHRDL1 was significantly downregulated in most tumor tissues of paired samples based on the data from TCGA database (Fig. 2C, *P* < .001).

**Figure 3.**
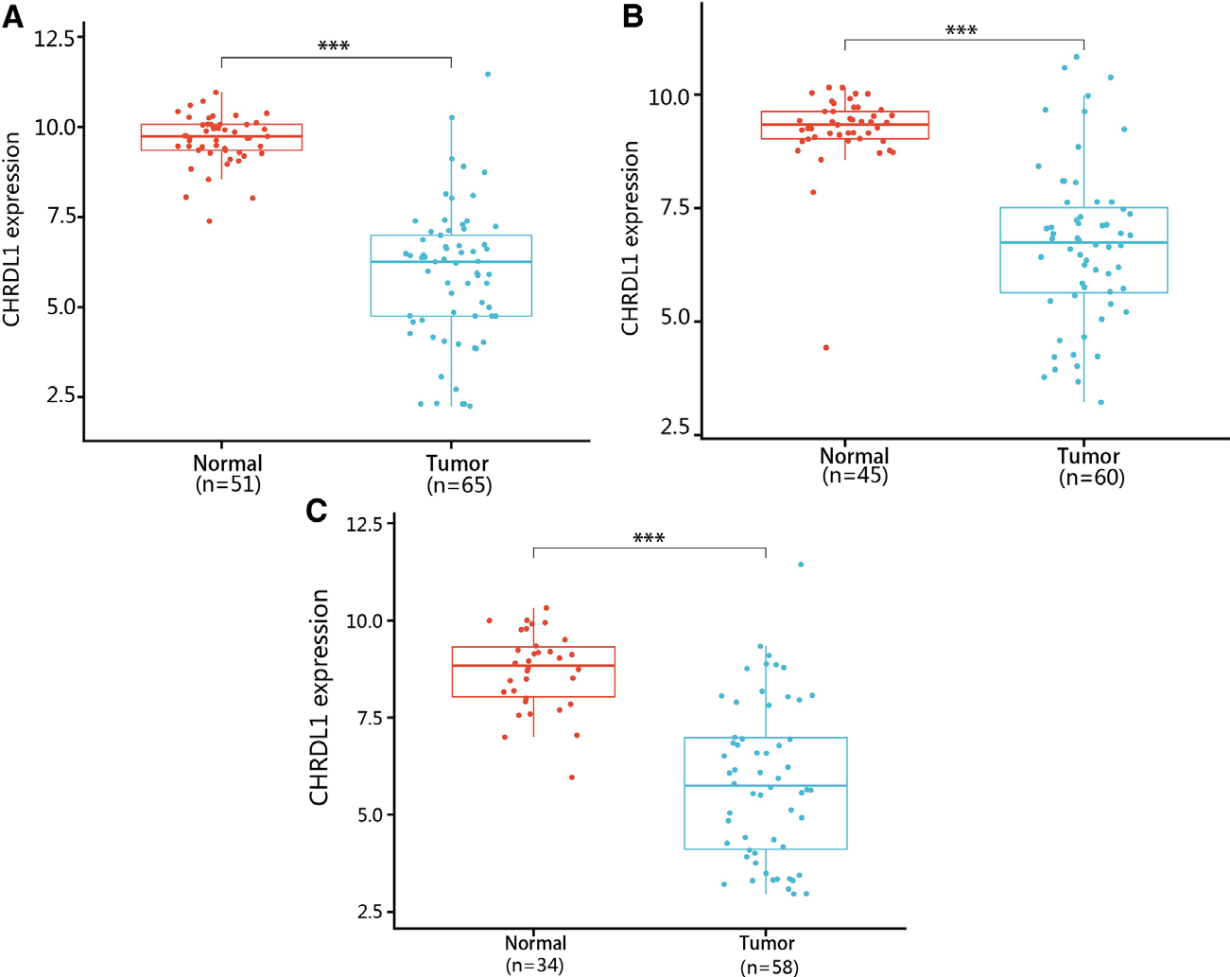
The expression of CHRDL1 in THCA and normal tissues was confirmed based on the GEO datasets including (A) GSE33570, (B) GSE33630, and (C) GSE60542. ^***^P < .001. CHRDL1 = chordin-like 1, GEO = Gene Expression Omnibus, THCA = thyroid cancer.

Then, GSE33570, GSE33630 and GSE60542 datasets were used for confirming the change of CHRDL1 expression in THCA tissues. The results showed that CHRDL1 expression levels were also notably downgraded in THCA tissues compared with normal tissues (Fig. 3A–C, *P* < .001).

**Figure 4.**
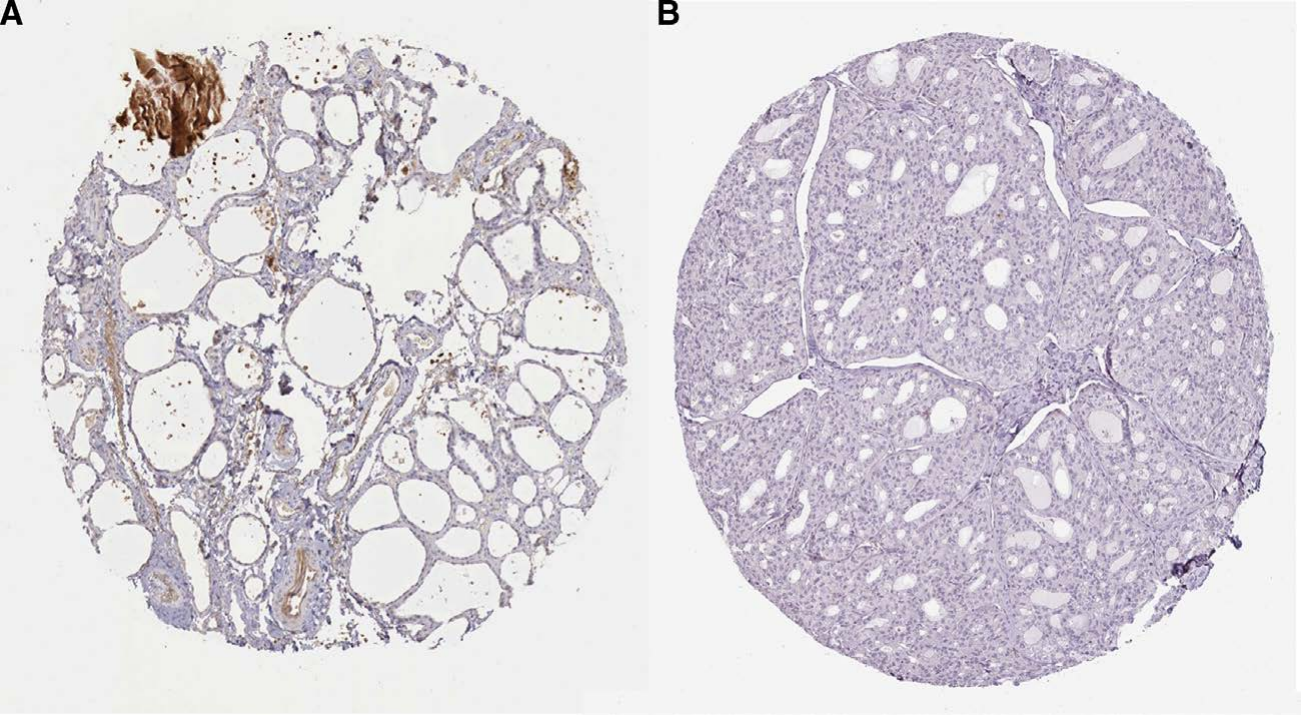
The protein expression of CHRDL1 in thyroid cells. (A) The normal thyroid glandular cell was obtained from a 76-years-old man. (B) The thyroid glandular cancer cell was obtained from a 75-year-old man. CHRDL1 = chordin-like 1.

To further identify the CHRDL1 expression in THCA and normal tissues, we analyzed the IHC results of CHRDL1 in THCA and normal tissues. These images also showed that the protein expression of CHRDL1 was clearly downregulated and even not detected in THCA cells (Fig. 4, *P* < .001).

**Figure 5.**
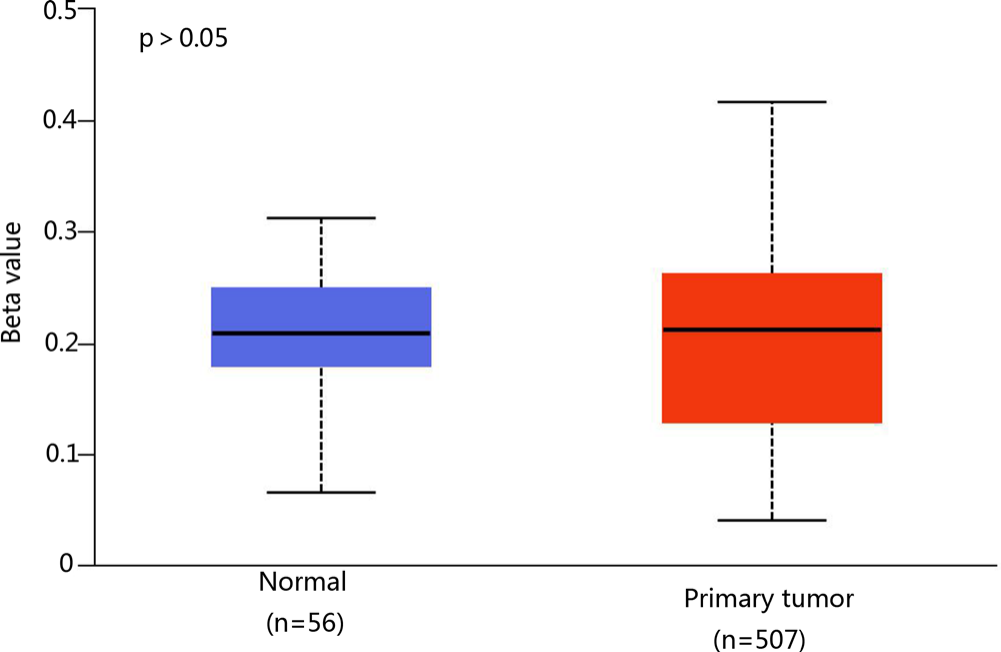
The comparison of CHRDL1 methylation levels between normal and THCA tissues according to the data from TCGA. The results of CHRDL1 methylation level were presented with a beta value which indicates the level of DNA methylation ranging from 0 (unmethylated) to 1 (fully methylated). CHRDL1 = chordin-like 1, TCGA = the cancer genome atlas, THCA = thyroid cancer.

As the methylation plays a vital role in THCA development and mRNA expression level,^[22,23]^ we analyzed the methylation level of CHRDL1 through the data of TCGA. However, there was no difference in the CHRDL1 methylation level between THCA and normal tissues (Fig. 5).

### 3.2. The correlation between CHRDL1 expression and clinicopathology in THCA

**Figure 6.**
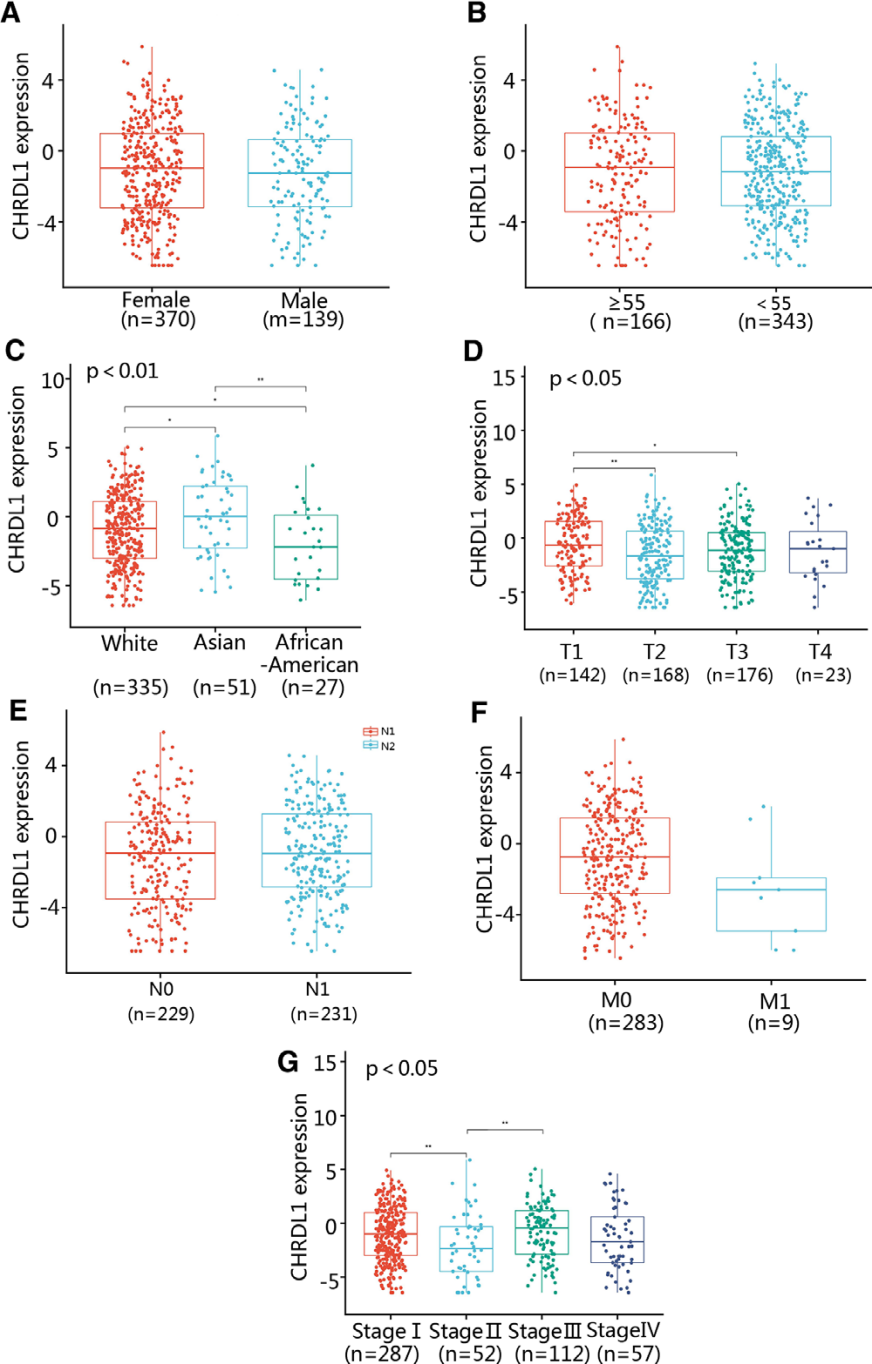
Box plots determined CHRDL1 expression in different groups of patients based on clinical data from the TCGA datasets. Analysis was shown for (A) gender, (B) age, (C) race, (D) T stages, (E) N stages, (F) M stages, and (G) pathologic stages. ^*^P < .05, ^**^ *P* < .01, ^***^P < .001. CHRDL1 = chordin-like 1, TCGA = the cancer genome atlas.

To assess the relationship between the CHRDL1 expression and clinicopathological features in THCA, we determined the CHRDL1 expression in different gender, ages, races, T stages, N stages, M stages and pathologic stages (Fig. 6A–G). As shown in Figure 6A,B,E,F, some factors containing gender, age, N stage and M stage were not related to CHRDL1 expression. However, CHRDL1 expression in Asian was markedly upregulated compared with White and African-American, and the expression level of CHRDL1 in White was also remarkably higher than in African-American (Fig. 6C, *P* < .01). Besides, the CHRDL1 expression level was obviously increased in the *T*1 stage compared with *T*2 and *T*3 stages, while there was no significant difference in CHRDL1 expression between *T*2 and *T*3 stages (Fig. 6D, *P* < .05). Moreover, the CHRDL1 expression level in stage II was dramatically downgraded compared with stage I and stage III (Fig. 6G, *P* < .05).

### 3.3. The correlation between CHRDL1 expression and prognosis in THCA

**Figure 7.**
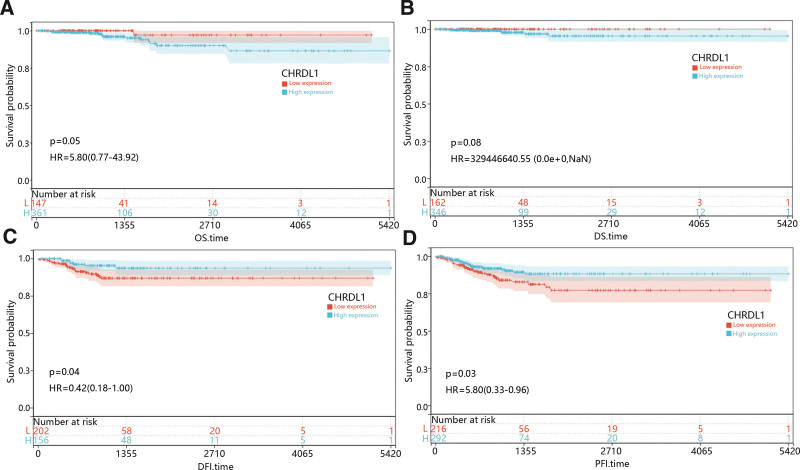
K–M survival curves compared the high and low expression of CHRDL1 in THCA. Survival curves of (A) OS, (B) DSS, (C) DFI, and (D) PFI time between high and low CHRDL1 expression in patients with THCA. The red lines represented low expression of CHRDL1, while blue lines represented high expression of CHRDL1. CHRDL1 = chordin-like 1, DFI = disease-free interval, DSS = disease-specific survival, K–M = Kaplan–Meier, OS = overall survival, PFI = progression-free interval, THCA = thyroid cancer.

With the purpose of assessing the prognostic value of CHRDL1 expression in THCA, we studied the relationship between CHRDL1 expression and survival time including overall survival, disease-specific survival, DFI and PFI, and the results were presented in Figure 7A–D. K–M survival curves revealed that low expression of CHRDL1 was obviously associated with poor DFI and PFI time (Fig. 7C–D, *P* < .05).

Next, we used the univariate cox regression to determine the relationship between clinical characteristics and CHRDL1 with DFI and PFI, respectively. The results of univariate cox regression showed that there was a significant correlation between CHRDL1 expression and PFI time (Table 1, *P* = .036), but the correlation between CHRDL1 expression and DFI time was not close (*P* = .118, data not shown). Thus, the PFI time was chosen for the next research.

**Table 1:** The univariate and multivariate analyses of age, pathologic stage and CHRDL1 expression in PFI time.

		Univariate analysis		Multivariate analysis	
	Number (N)	HR (95% CI)	*P* value	HR (95% CI)	*P* value
Gender	509		.061		.395
Female	370	reference		reference	
Male	139	1.698 (0.977–2.951)		1.286 (0.720–2.297)	
Age	509				
≥55	166	reference		reference	
<55	343	0.450 (0.264–0.768)	.003	0.658 (0.342–1.265)	.209
Stage	508				
I	287	reference		reference	
II	52	1.364 (0.512–3.636)	.535	1.029 (0.362–2.928)	.957
III	112	2.196 (1.138–4.240)	.019	1.791 (0.852–3.765)	.124
IV	57	4.042 (2.006–8.145)	.000	2.937 (1.299–6.639)	.010
CHRDL1 expression	509				
High	292	reference		reference	
Low	217	1.775 (1.037–3.037)	.036	1.850 (1.075–3.185)	.026

CHRDL1 = chordin-like 1, PFI = progression-free interval.

**Figure 8.**
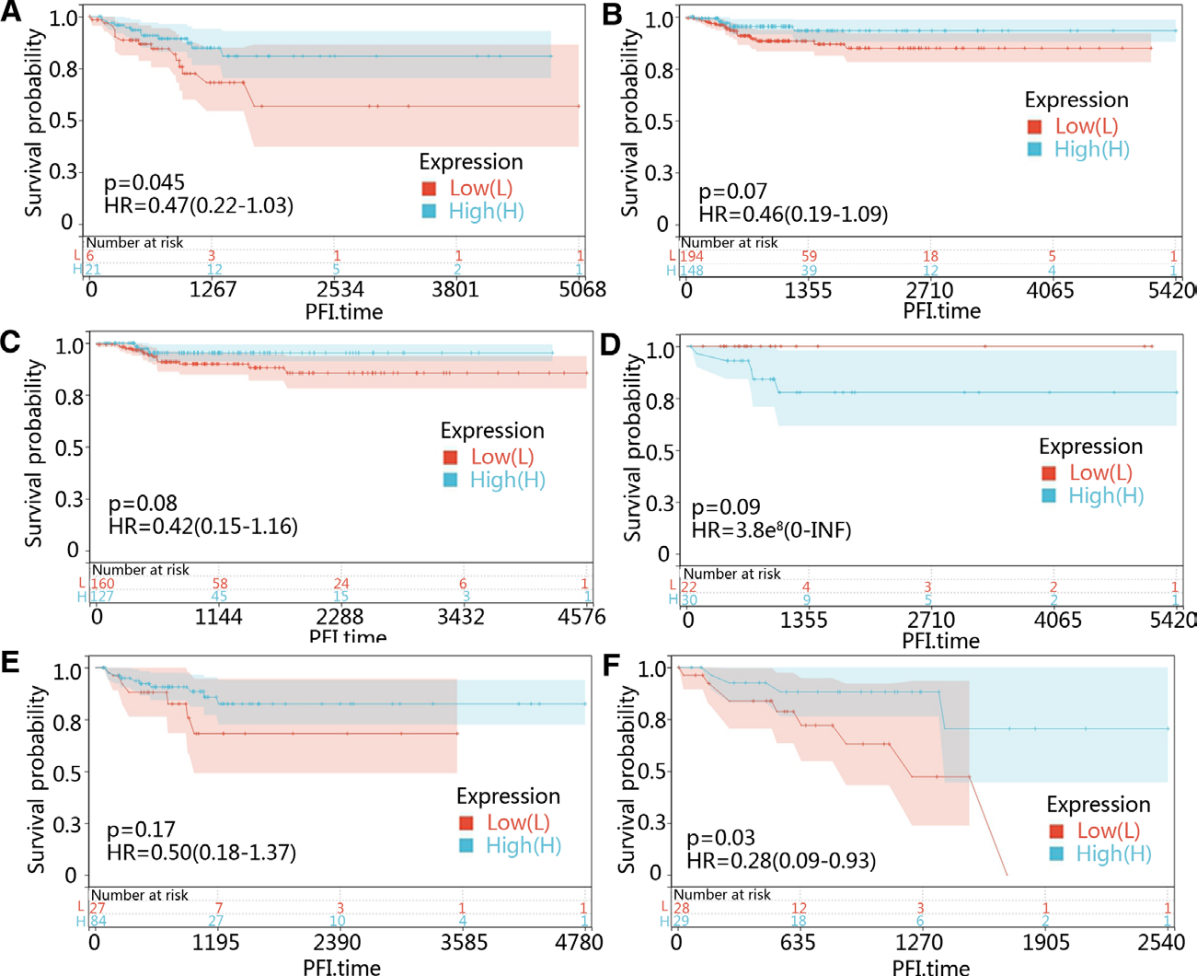
The effect of CHRDL1 expression level on PFI time under different clinical factors. The survival curves showed the effect of high-expressed and low-expressed CHRDL1 on PFI time in the patients (A) < 55 years old, (B) ≥ 55 years old, (C) in pathologic stage I, (D) pathologic stage II, (E) pathologic stage III, or (F) pathologic stage IV. The red lines represented low expression of CHRDL1, while blue lines represented high expression of CHRDL1. CHRDL1 = chordin-like 1, PFI = progression-free interval.

Except for the CHRDL1 expression, we also identified that younger age (*P* = .003) and advanced stages (*P* < .05) were memorably related to PFI time through univariate cox regression (Table 1). Therefore, we further performed the subgroup survival analysis on CHRDL1 in different age and stage groups. From Figure 8A–F, it was obvious that the low CHRDL1 expression was evidently related to the prognosis in the people under 55 years old (*P* = .045) and stage IV (*P* = .03).

Additionally, multivariable cox regression showed that pathologic stage (*P* = .029) and CHRDL1 expression (*P* = .023) were also clearly related to the PFI time (Table 1), which suggested that CHRDL1 expression and pathologic stage were independent predictors of PFI time in the patients diagnosed with THCA.

**Figure 9.**
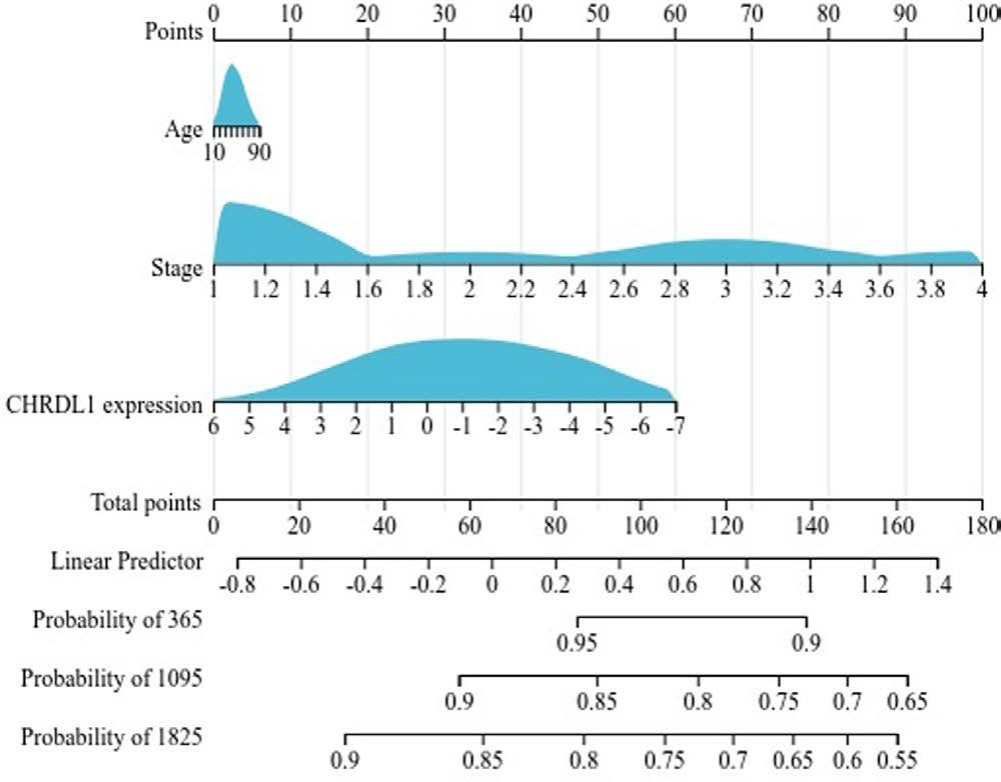
The nomogram model for predicting 1,3,5-year PFI in THCA patients. The length of the line in the nomogram represented the effect of corresponding variables on PFI time. The total points obtained by adding the scores of every factor could be used for assessing the probability of 1, 2, and 3-year PFI, PFI = progression-free interval, THCA = thyroid cancer.

Next, in accordance with the results of univariate cox regression, we used the R software package and integrated the data of PFI time, survival status and 3 characteristics, to establish a nomogram of 1, 3, and 5-year PFI time and evaluate the prognostic significance of these characteristics (Fig. 9). It was obvious that pathologic stage played an essential role in the PFI time of THCA patients. Moreover, CHRDL1 expression was second to the pathologic stage in importance to PFI time.

### 3.4. The GSEA analysis of CHRDL1 in THCA

**Figure 10.**
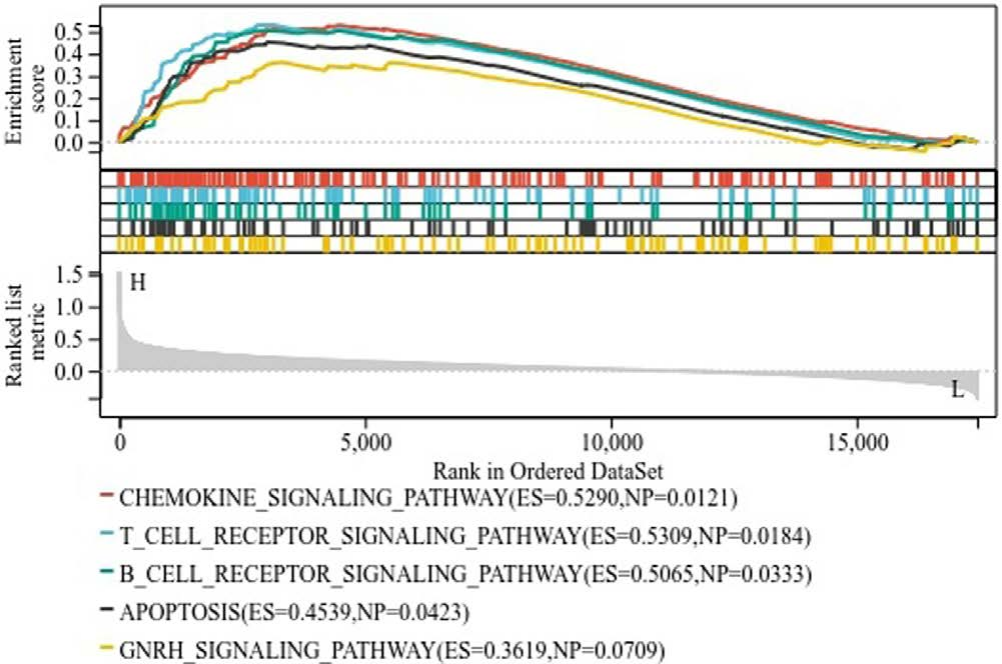
The correlation between CHRDL1-related genes and PFI time. Survival curves showed the effect of high-expressed and low-expressed (A) SCARA5, (B) CCL21, (C) PODN, (D) DPT, and (E) OSR1 on PFI time. The red lines represented low expression of CHRDL1, while blue lines represented high expression of CHRDL1. CCL21 = C-C motif chemokine ligand 21, CHRDL1 = chordin-like 1, DPT = dermatopontin, OSR1 = odd-skipped related transcription factor 1, PFI = progression-free interval, PODN = podocin, SCARA5 = scavenger receptor class A member 5.

Due to the significant clinical value of CHRDL1 in THCA, this study further explored the potential regulatory mechanism associated with CHRDL1. The patients were divided into high-expressed group and low-expressed group according to the expression of CHRDL1 and then GSEA analysis was performed to predict the enriched pathways. GSEA analysis indicated that 137 pathways were enriched in the high-expressed CHRDL1 group and 50 pathways were enriched in the low-expressed CHRDL1 group. The 5 main downregulated pathways were presented in Figure 10, containing chemokine signaling pathway, T cell receptor signaling pathway, B cell receptor signaling pathway, apoptosis and gonadotropin-releasing hormone receptor signaling pathway.

### 3.5. Immune cell infiltration

**Figure 11.**
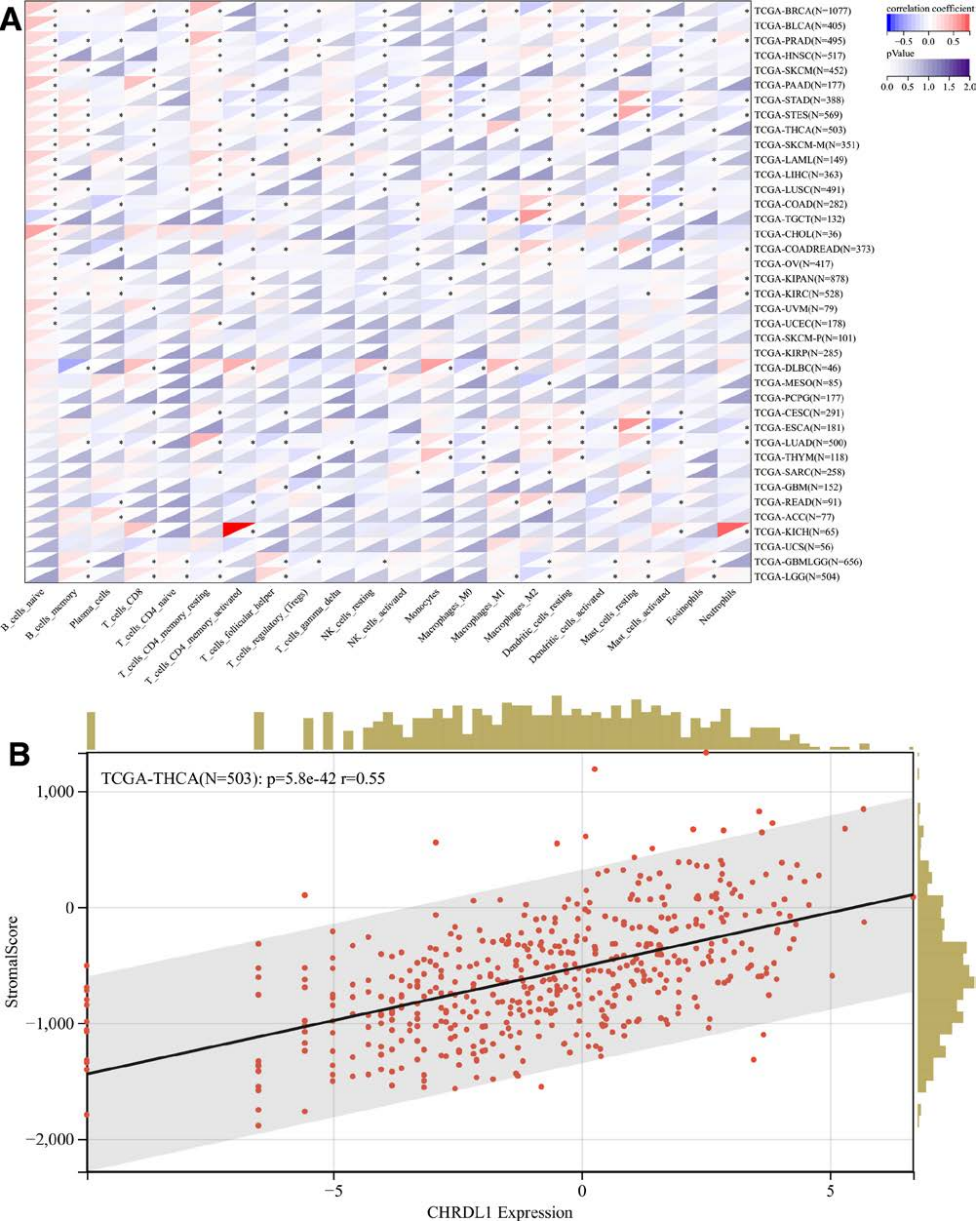
The correlation between PFI time and the expression of CHRDL1 co-expressed genes, including (A) SCARA5, (B) CCL21, (C) PODN, (D) DPT, and (E) OSR1. The red line indicated that these genes and CHRDL1 were co-upregulated. The green line indicated that these genes and CHRDL1 were co-downregulated. CCL21 = C-C motif chemokine ligand 21, CHRDL1 = chordin-like 1, DPT = dermatopontin, OSR1 = odd-skipped related transcription factor 1, PFI = progression-free interval, PODN = podocin, SCARA5 = scavenger receptor class A member 5.

Previous studies identified that immune cell infiltration played a role in the survival time of patients,^[24]^ and GSEA results showed that CHRDL1 enriched in T and B cell receptor signaling pathways, which were associated with the immune system, so a pan-cancer analysis of various immune cells was performed. As shown in Figure 11A. it was worth noting that the CHRDL1 expression was positively correlated to the infiltration levels of dendritic cells resting, macrophages M1, T cells follicular helper, T cells CD4 memory resting, B cells memory and B cells naïve in THCA, but negatively correlated to eosinophils, mast cells resting, monocytes, natural killer cells resting, T cells regulatory and T cells CD8 (*P* < .05).

Next, we explored whether CHRDL1 was involved in immune infiltration in THCA or not. Figure 11B exhibited that CHRDL1 expression had a significant positive correlation with immune infiltration in THCA (*P* < .001).

### 3.6. The correlation of CHRDL1-related gene expressions with prognosis

**Figure 12.**
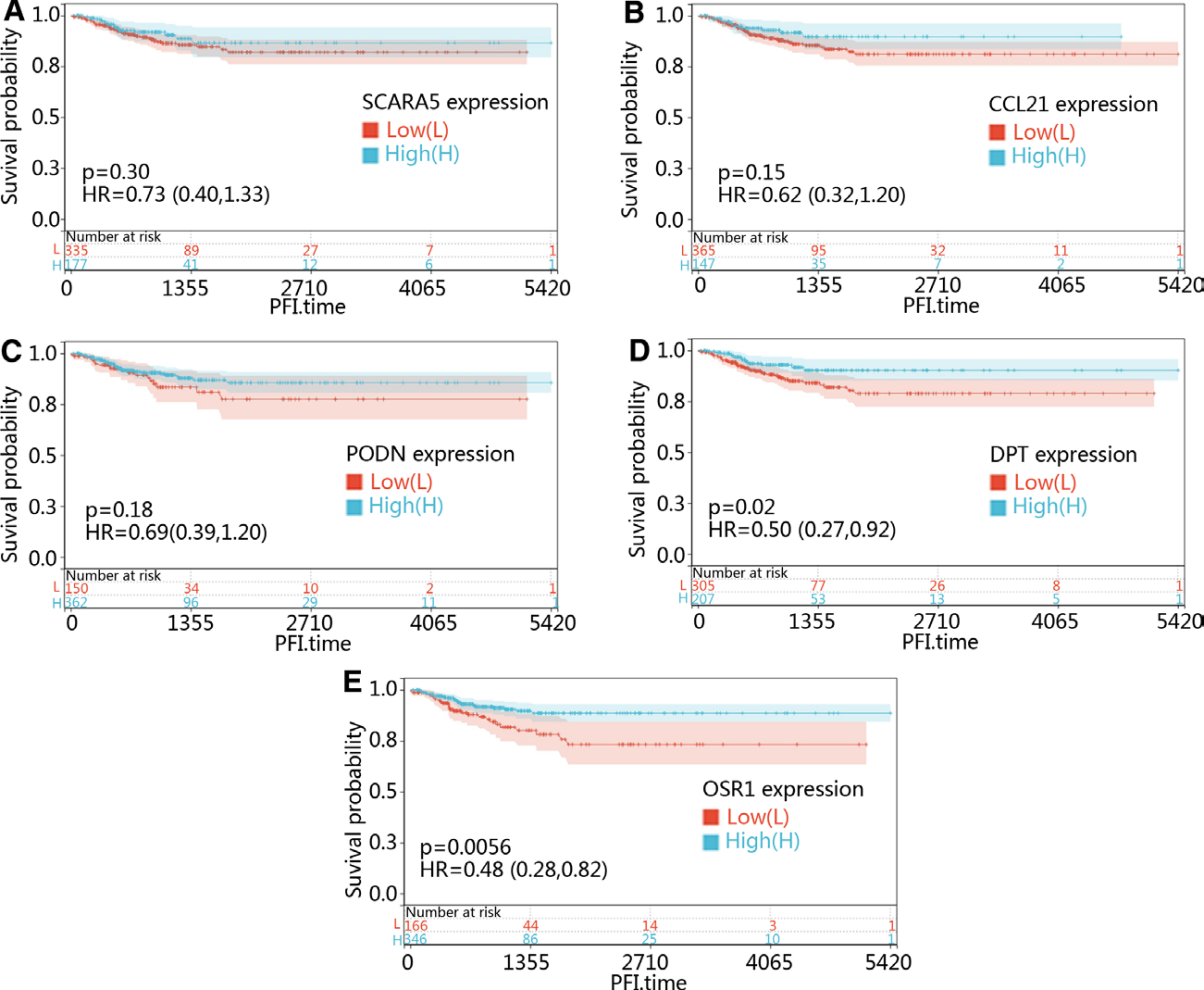
The functional pathways of CHRDL1 in THCA. The red line indicated the chemokine signaling pathway; the blue line indicated the T cell receptor signaling pathway; the green line indicated the B cell receptor signaling pathway; the black line indicated the apoptosis; the yellow line indicated the Gonadotropin-releasing hormone (GnRH) receptor signaling pathway. CHRDL1 = chordin-like 1, GnRH = gonadotropin-releasing hormone, THCA = thyroid cancer.

Additionally, the function of most mRNA was usually affected by other genes, so we try to identified the role of the co-expressed gene of CHRDL1 in THCA. We used the cBioPortal online platform to predict CHRDL1 co-expressed genes within the TCGA pan-cancer atlas. After filtering the prediction results, the positively correlated genes with Pearson correlation coefficient > 0.5 were selected, and. After the top 5 genes positively correlated to the CHRDL1 in THCA, including scavenger receptor class A member 5, dermatopontin (DPT), C-C motif chemokine ligand 21 (CCL21), podocin and odd-skipped related transcription factor 1 (OSR1) were selected, we first explore the function of them in PFI of THCA. As shown in Figure 12A–E, low expression levels of DPT (*P* = .02) and OSR1 (*P* = .0056) caused a poor prognosis compared with the high expression levels.

**Figure 13.**
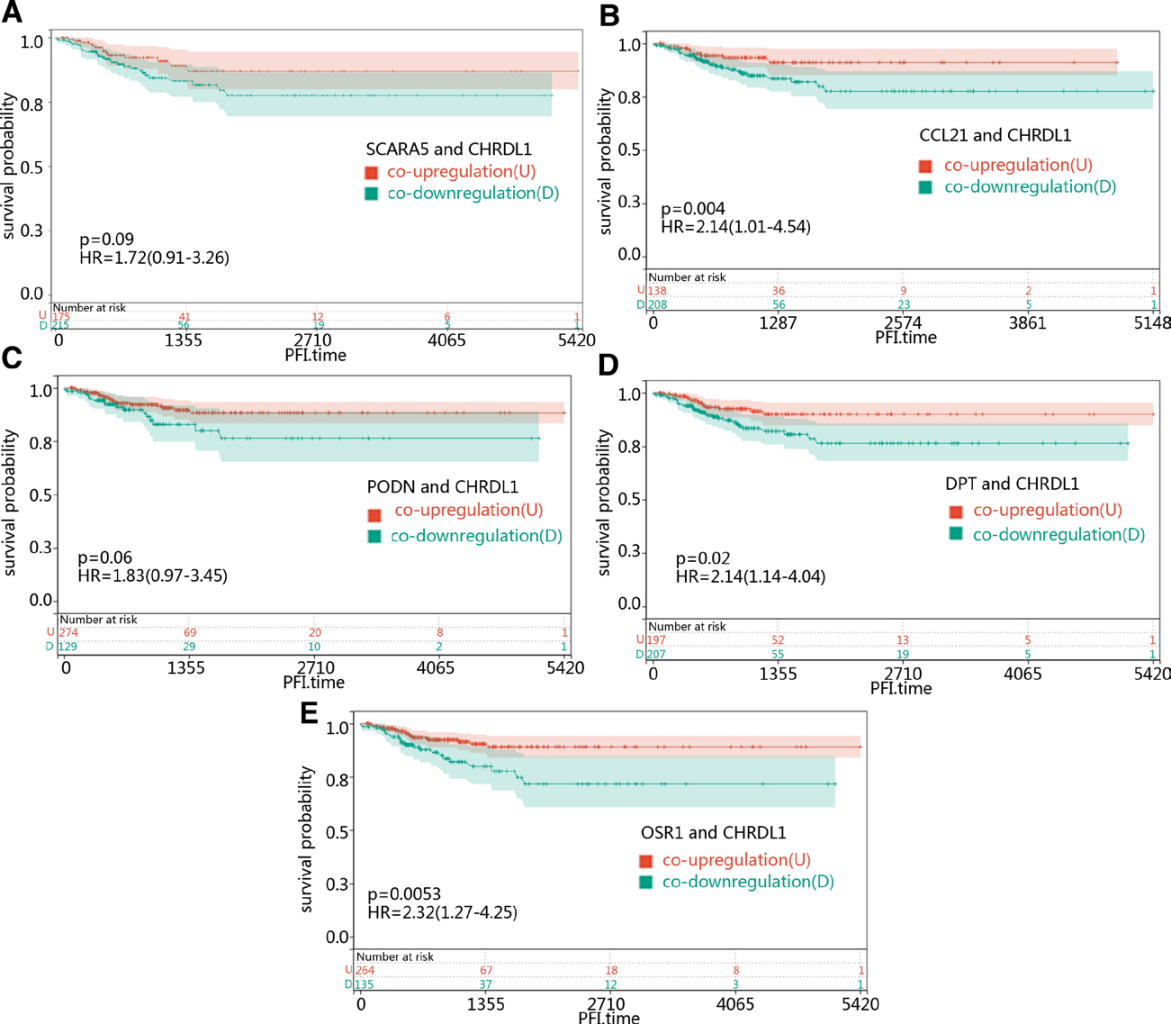
GO and KEGG enrichment analysis of CHRDL1 and its co-expressed genes. The GO enrichment analysis was shown for (A) BP, (B) CC, and (C) MF. (D) The main function of CHRDL1 was analyzed by KEGG enrichment analysis. The X-axis was the value of the gene ratio in the term and the Y-axis was the name of the terms. The size of the circles indicated the number of genes enriched in the term, and the color of the circle indicated the *P*-value. BP = biological processes, CC = cellular component, CHRDL1 = chordin-like 1, GO = Gene Ontology, KEGG = Kyoto Encyclopedia of Genes and Genomes.

Then, to further explore the combined effect of CHRDL1 and its co-expressed genes in PFI time in THCA, all the patients were divided into co-upregulation and co-downregulation groups according to expression level of CHRDL1 and its co-expressed genes. From Figure 13A–E, it could be seen that the co-downregulation group of CCL21 (Fig. 13B, *P* = .004), DPT (Fig. 13D, *P* = .02) and OSR1 (Fig. 13E, *P* = .0053) were clearly associated to a poor prognosis compared with the co-upregulation group.

### 3.7. The enrichment analysis of CHRDL1 and co-expressed genes in THCA

**Figure 14.**
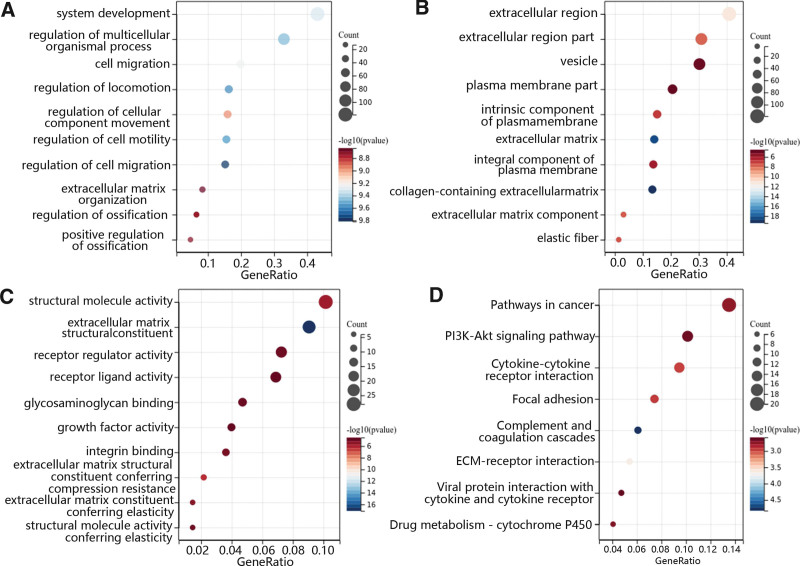
The immune infiltration in cancers. (A) The association between 22 cancers and 39 immune cells was based on the data derived from the TCGA Pan-Cancer dataset. (B) The correlation of CHRDL1 expression and immune infiltration in THCA. ^*^P < .05. CHRDL1 = chordin-like 1, TCGA = the cancer genome atlas, THCA = thyroid cancer.

To determine CHRDL1 co-expressed genes and their function, the top 100 correlated genes were selected 5 according to the above results. Then, the GO and KEGG analyses were performed to determine the function of CHRDL1 and its co-expressed genes. In terms of biological processes, GO enrichment analysis revealed that CHRDL1 and its co-expressed genes were mainly enriched in the system development, cell migration, extracellular matrix organization and regulation of cellular movement and development (Fig. 14A). In the aspect of cellular component, these genes were enriched in the extracellular region, elastic fiber and the component of the plasma membrane (Fig. 14B). Besides, the main enriched molecular function included extracellular matrix structural constituent, binding of integrin and glycosaminoglycan, and the activity of structural molecule, receptor regulator, receptor-ligand and growth factor (Fig. 14C). Furthermore, the results of KEGG pathway enrichment analysis further demonstrated that CHRDL1 and its co-expressed genes were enriched in the pathway in cancer, phosphatidylinositol 3-kinase (PI3K)-AKT signaling pathway, cytokine-cytokine receptor interaction, focal adhesion and others (Fig. 14D).

## 4. Discussion

Previous studies had identified that the expression of CHRDL1 was abnormal in many cancers including colorectal adenoma, GC and lung adenocarcinoma.^[11,25,26]^ Additionally, CHRDL1 was considered as a prognosis predictor of lung adenocarcinoma and GC.^[26,27]^ In our study, we found that the mRNA expression and protein expression levels of CHRDL1 were downgraded in THCA cells compared with normal cells. Besides, we found that low mRNA expression caused a poor prognosis, which suggested that low expression of CHRDL1 promoted the development of THCA. Moreover, our results highlighted that CHRDL1 expression was an independent predictor of PFI in THCA through univariate and multivariable cox regression, which indicated that CHRDL1 expression could be a potential biomarker of THCA. However, the mechanism of CHRDL1 in THCA was not reported.

Many studies identified that the development of THCA was accompanied by DNA methylation,^[28–30]^ and Pei et al claimed that the hypermethylation of CHRDL1 promoter promoted the combination of BMP and BMP receptor II to induce the proliferation and migration of GC cells though downregulating mRNA expression of CHRDL1.^[11]^ However, our results showed that there was no marked difference in CHRDL1 methylation level between THCA and normal samples, which suggested that CHRDL1 methylation may not be involved in the THCA development.

In addition, the prognosis of cancer patients was affected by the combined action of many genes.^[31]^ Morillo-Bernal et al^[32]^ found that silencing Forkhead Box E1inhibited the migration and invasion of THCA cells through downregulating ZEB1 expression. Furthermore, Cyr-Depauw et al^[17]^ claimed that CHRDL1 could inhibit migration and invasion of BC cells through inhibition of BMP4 expression in BC. Nevertheless, the function of CHRDL1 in THCA was unclear. To further study the underlying mechanisms of CHRDL1 expression in THCA, we determined the co-expressed genes of CHRDL1 followed by the correlation analysis of co-expressed genes and prognosis. The results manifested that the co-expressed genes of CHRDL1 included scavenger receptor class A member 5, CCL21, podocin, DPT and OSR1. Previous studies determined that these genes were involved in the development of various cancers through regulating cell proliferation and invasion.^[33–39]^ What’s more, the DPT and OSR1 expressions significantly affected the prognosis, but CCL21 expression only obviously affected prognosis when it was co-downregulated with CHRDL1. Moreover, Luo et al identified that co-expression of CCL21 and interleukin 7 enhanced the inhibiting function of cancer development.^[40]^ These results suggested that the function of CCL21 in THCA development depended on the co-expression with CHRDL1.

The prognosis was also affected by the combined action of signal pathways.^[31]^ Wu et al^[41]^ identified that silencing CHRDL1 could promote the migration of oral squamous cell carcinoma cells through activating the mitogen-activated protein kinase signal pathway. Besides, the reduced CHRDL1 expression also caused the poor prognosis of GC through activating extracellular regulated protein kinases and Akt signal pathways to promote the proliferation and migration of GC cells.^[11]^ Combined our results of GO and KEGG enrichment analysis, we found the CHRDL1 and its co-expressed genes were mainly involved in the signal pathway related to the development of cancer, such as cell migration, PI3K-Akt signaling pathway and extracellular matrix (ECM)-receptor interaction.^[42–46]^ It confirmed that CHRDL1 could inhibit the development of THCA through some signal pathways.

On the other hand, the GSEA pathway enrichment analysis showed that CHRDL1 were mainly enriched in the chemokine signal pathway, T cell receptor signal pathway, B cell receptor signal pathway, apoptosis and gonadotropin-releasing hormone signal pathway. Obviously, these signal pathways were closely related to the immune cell. It suggested that the function of CHRDL1 in THCA may have a concern with the immune cells.

The immune cell is an important part of the tumor immune microenvironment and plays a role in the development of cancer.^[47]^ In THCA, the responses of innate immune cells and adaptive immune cells were dependent on many factors including chemokines.^[48]^ In fact, the genes co-expressed with CHRDL1, namely, CCL21, DPT and OSR1 were related to immune cell infiltration except for the function of inhibiting cell proliferation and migration.^[38,49–51]^ Moreover, our results of immune infiltration analysis demonstrated that the function of CHRDL1 was enriched in the chemokines signal pathway and CHRDL1 was positively or negatively correlated to immune cells containing T cells and B cells, respectively. These results suggested that CHRDL1 inhibited cancer development through regulating the immune microenvironment.

In conclusion, we identified that CHRDL1 expression level was downregulated in THCA, which was associated to poor prognosis of PFI time for patients diagnosed with THCA. Besides, CHRDL1 may be involved in some signal pathway related to the cancer development and immune response, which indicated CHARDL1 may be a potential target of treatment. However, some problems still not be solved in this article. For example, we found that CHRDL1 also promoted the PI3K-Akt signal pathway which promoted the development of cancer. It contradicts the function of CHRDL1 which is regarded as an anti-oncogene. The specific mechanism of CHRDL1 in THCA need to be verified through further experiment. But our study still provides a new sight and some basic theoretical basis for exploring the mechanism of THCA development.

## Author contributions

**Conceptualization:** Jia-Wei Yu, Jie-Wu Zhang.

**Data curation:** Jia-Wei Yu, Jie-Wu Zhang.

**Formal analysis:** Jia-Wei Yu, Rui Pang, Bo Liu.

**Investigation:** Rui Pang, Bo Liu, Liang Zhang.

**Methodology:** Jia-Wei Yu, Rui Pang, Bo Liu, Liang Zhang, Jie-Wu Zhang.

**Resources:** Bo Liu, Liang Zhang.

**Supervision:** Bo Liu.

**Validation:** Jia-Wei Yu.

**Visualization:** Rui Pang, Liang Zhang, Jie-Wu Zhang.

**Writing – original draft:** Jia-Wei Yu, Liang Zhang, Jie-Wu Zhang.

**Writing – review & editing:** Rui Pang, Bo Liu.
